# Association between BRINDA-corrected iron metabolism and lung function in the community: the CoLaus|PneumoLaus study

**DOI:** 10.1186/s12890-025-03810-x

**Published:** 2025-09-30

**Authors:** Brice Touilloux, Alessio Casutt, Minh Khoa Truong, Cédric Bongard, Benoit Lechartier, Pedro Marques-Vidal, Peter Vollenweider, Julien Vaucher, Christophe Von von Garnier

**Affiliations:** 1 Division of Pulmonology, Department of Medicine, Lausanne University Hospital (CHUV), University of Lausanne (UNIL), Lausanne, Switzerland; 2 Division of Pulmonology, Department of Medicine and Specialties, Fribourg Hospital and University of Fribourg, Fribourg, Switzerland; 3 Division of Pulmonology, Ospedale Regionale di Lugano, Ente Ospedaliero Cantonale, Università della Svizzera Italiana (USI), Lugano, Lugano, Switzerland; 4 Division of Internal Medicine, Department of Medicine, Lausanne University Hospital (CHUV), University of Lausanne (UNIL), Lausanne, Switzerland; 5 Division of Internal medicine, Department of Medicine and Specialties, Fribourg Hospital and University of Fribourg, Fribourg, Switzerland

**Keywords:** Spirometry, Lung function, Iron, Metabolism, Transferrin, Ferritin, Small airways

## Abstract

**Background:**

Iron metabolism and its relationship to lung volumes remains poorly understood, with inconsistent findings reported across the literature. We analysed the association of markers of inflammation and iron metabolism with lung function in a large population-based cohort.

**Methods:**

PneumoLaus is a sub-study of CoLaus|PsyCoLaus, an ongoing prospective observational study conducted in Lausanne, Switzerland. Within PneumoLaus, participants performed spirometry and blood sampling at two time points (baseline, 2014–2017; follow-up (FU), 2018–2021). The associations between ferritin and transferrin using BRINDA correction with spirometric values were assessed by Pearson correlation. We performed multivariable linear regressions using spirometry values as dependent variable adjusted for main confounders.

**Results:**

3102 (women, 56%) and 1989 (women, 55%) participants were included at baseline and FU, respectively. In both surveys, ferritin levels were not associated with FEV1, FVC and MMEF, even using the adjusted model. A weak negative association was observed only at FU in women with FEV1 and FVC. In both surveys, transferrin was negatively associated with FEV1 and FVC (standardised β coefficients −0.061 to −0.102, *p* < 0.001) in all subjects and in women. In the adjusted model, FEV1 is reduced by 47 to 59 mL and FVC by 63 to 71 mL for 1-SD increase of transferrin levels. Transferrin saturation was not associated with spirometric values.

**Conclusions:**

In this population-based cohort, we observed no reproductible association between ferritin levels and spirometric values. However, transferrin levels were negatively associated with FEV1 and FVC, suggesting that iron metabolism, particularly the requirement for iron in the organism, is linked to reduced lung volumes.

**Trial registration:**

Not required. This study is not a clinical trial. This is a population based observational study. The approval number reference 16/03 13403,13405bis, 134052to5 addenda 1to4. For other approvals, see the section dedicated to page 26 and 27.

**Supplementary Information:**

The online version contains supplementary material available at 10.1186/s12890-025-03810-x.

## Summary

We did not observe a reproductible association between ferritin and spirometric values. Transferrin levels were negatively associated with FEV1 and FVC, but not consistently with MMEF. Iron metabolism, particularly the requirement for iron in the organism, could be linked to reduced lung volumes.

## Background

Multiple indicators suggest an association between iron metabolism and lung function, but underlying mechanisms remain poorly understood and the evidence in the literature is conflicting. A study including over 42,927 healthy Korean males observed a significant association between hyperferritinemia and decreased lung function as measured by Forced Vital Capacity (FVC) and Forced Expiratory Volume in 1 s (FEV1) in % predicted [1]. Another Korean study reported a negative association between ferritin levels and FVC as % of predicted in postmenopausal women [2]. Brigham et al. [3] found that higher ferritin was associated with lower FVC in young and middle-aged women. A genetic study using mendelian randomisation principles suggested a very weak positive association between ferritin and lung volume, and a very weak negative association between lung volume and transferrin [4]. A recent study in children and adolescents with β-thalassemia major found a lower FVC in the high ferritin group compared to the low ferritin group [5].

This negative association differed from other studies that found a positive relationship between ferritin and lung function [4, 6, 7]. A study conducted on stable Chronic Obstructive Pulmonary Disorder (COPD) patients also found that lower ferritin levels were associated with a greater decline in FEV1 over time, suggesting that iron deficiency could play a role in the disease-progression [8].

Both iron overload and deficiency may affect lung function by modulating innate and adaptive immunity [9]. Cigarette smoking induces pathogenic changes in lung cells, including dysregulation of iron homeostasis [10]. Smoking causes iron overload and alveolar macrophages dysfunctions. Serum ferritin, as well as transferrin, is higher in smokers compared to non-smokers [11]. It is proposed that iron accumulation in smokers triggers a biochemical cascade resulting in injury, notably airway obstruction [7]. Even though evidence points towards a deleterious effect of iron accumulation on lung function, results are conflicting and should be replicated using a large panel of markers of iron metabolism with various adjustments and corrections.

In the present study, we investigated the association of markers of iron metabolism with lung function in an urban middle-aged population and population based observational cohort.

## Methods

### Setting and selection of participants

The PneumoLaus study and methodology has been previously described [12, 13]. Briefly, PneumoLaus is a sub-study of the CoLaus|PsyCoLaus study (www.colaus-psycolaus.ch), a prospective ongoing population-based study in Lausanne, Switzerland. The baseline survey were assessed between June 2014 and August 2017 and the follow-up (FU) survey between June 2018 and February 2021. Blood tests and other measures were recorded during CoLaus|PsyCoLaus. The ethics commission (PB_2018-00038, reference 239/09) and all participants provided written informed consent.

### Spirometric manoeuvres and respiratory risk factors

The PneumoLaus methodology was already described [13]. Each spirometric manoeuvre was automatically scored using computer-based acceptability and reproducibility criteria according to the international standards [14, 15]. The maximal mid expiratory flow (MMEF) was defined by the mean forced expiratory flow between 25% and 75% of the FVC. The GLI-2012 reference values were utilised [16]. FEV1, FVC and MMEF were expressed in absolute values and z-scores.

### Blood tests

Plasma ferritin, transferrin and high-sensitivity C-reactive protein (hs-CRP) levels were measured by blood analysis during baseline and FU of PneumoLaus. Transferrin saturation (TSAT) was also measured at FU PneumoLaus visits. Clinical chemistry tests were performed at the central laboratory of the Lausanne University Hospital (CHUV). Iron was measured by colorimetric method (ferrozine, BioSystems); ferritin (µg/L) by immunoturbidimetric method (Tina-quant 4th generation, Roche Diagnostics, Switzerland), and transferrin (µmol/L) by immunoassay. Transferrin saturation (TSAT, %) was determined as (serum iron ÷ (25 × transferrin)) × 100 [17].

### Covariates

Educational level was categorised into four groups: compulsory school, apprenticeship, high school, university degree. A face-to-face structured interview by the respiratory practitioner on the day of spirometry. Smoking status was categorised as current, former, or never smoker.

### Inclusion and exclusion criteria

Participants from the PneumoLaus study (2014–17 and 2018–21) were considered as eligible. We excluded subjects with non-interpretable spirometry. Subjects without analysed blood tests and covariates were excluded.

### Statistical analyses

Participant characteristics are expressed as numbers (percentages) for categorical variables, and as means with standard deviation (SD) for continuous variables. Comparisons between-group were performed using Mann-Whitney or student’s t-test for dichotomous outcome variables.

Plasma ferritin levels, transferrin levels and transferrin saturation were adjusted by Biomarkers Reflecting Inflammation and Nutrition Determinants of Anemia (BRINDA) correction using hs-CRP only, due to the interaction between ferritin, transferrin and transferrin saturation with inflammation, even in mild inflammation [18–21]. Ferritin retained its logarithmic correction (used in the BRINDA correction) due to its skewed distribution. Transferrin was then converted back to its original unit.

Bivariate analyses between blood values and spirometric values were performed using Pearson correlation. We also performed a Spearman rank correlation analysis as a complementary analysis. Multivariable analyses were conducted using multiple linear regression, with each spirometric value analysed separately (in absolute value and z-score) as the dependent variable, and blood values (ferritin and transferrin in the first model, and ferritin, transferrin, TSAT in the second model used for FU only) as independent variables. The multivariable analyses were adjusted for age, smoking status, sex, BMI (as a continuous variable) and education level (as an ordinal variable). Results were firstly expressed as standardised beta (β) coefficients, corresponding to a change of one SD in the dependent variable for a change of one SD in the independent variable. Secondly, blood values were standardised, and results were expressed in mL for FEV1 and FVC per 1-SD increase in transferrin level and TSAT. Due to its logarithmic transformation, this 1-SD increase was not reported for ferritin. Each dependent variable was included in the models separately. The BRINDA adjusted values of log-transformed serum ferritin were closer to a normal distribution than the original values. All analyses were also stratified by sex.

Statistical significance is considered for a two-sided test with *p* < 0.01. Stata™ software (version 17.0, StataCorp, College Station, TX, USA) was used for statistics.

## Results

**Fig. 1:**
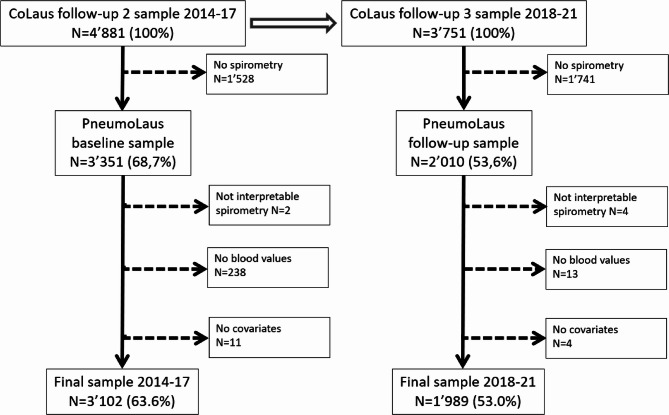
Participants selection

Of the initial 4881 participants of the baseline survey, 3102 subjects (56% women, age 62.8 ± 10.0 years) were included in the first analysis. Of the original 3751 participants of the FU survey, 1989 subjects (55% women, age 66.2 ± 9.5 years) were included in the second analysis (Fig. 1). Reasons for exclusion are summarised in Fig. 1. The characteristics, spirometry values and blood values of the participants at baseline and FU are summarised in Table 1 and S1 in the supporting information.

**Table 1:** Participants characteristics at baseline (2014-17) and FU (2018-21)

	Baseline (*n* = 3102)	Follow-up (*n* = 1989) *
Female sex, n (%)	1736 (56.0)	1101 (55.4)
Age in years, mean (+/-SD)	62.8 (10.0)	66.2 (9.5)
BMI, mean (+/-SD)	26.55 (4.65)	26.28 (4.50)
Caucasian, n (%)	3035 (97.8)	1950 (98.0)
Spirometric values		
FEV1 in Litre, mean (SD)	2.83 (0.80)	2.77 (0.76)
FEV1 in z-score, mean (SD)	+ 0.01 (1.07)	+ 0.11 (1.01)
FVC in Litre, mean (SD)	3.68 (0.98)	3.59 (0.96)
FVC in z-score, mean (SD)	+ 0.08 (0.95)	+ 0.14 (0.91)
MMEF in Litre/s, mean (SD)	2.57 (1.09)	2.51 (1.04)
MMEF in z-score, mean (SD)	+ 0.14 (1.18)	+ 0.29 (1.17)
Smoking status		
Former, n (%)	1014 (32.7)	623 (31.3)
Current, n (%)	559 (18.0)	319 (16.0)
Education level		
Compulsory school, n (%)	457 (14.7)	256 (12.9)
Apprenticeship, n (%)	1126 (36.3)	732 (36.8)
High school, n (%)	853 (27.5)	571 (28.7)
University, n (%)	666 (22.5)	430 (21.6)
hs-CRP in mg/L, mean (SD)	2.19 (3.18)	2.30 (3.13)
hs-CRP in mg/L, median (Q1-Q3)	1.1 (0.5–2.4)	1.3 (0.7–2.6)
Ferritin in µg/L, mean (SD)	183.7 (157.4)	176.5 (135.6)
Ferritin in µg/L, median (Q1-Q3)	148.0 (89.0–225.0)	146.0 (92.0–218.0)
Transferrin in µmol/L, mean (SD)	31.0 (4.2)	30.73 (4.07)
Transferrin saturation in %, mean (SD)	-	29.8 (9.5)

*MMEF* maximal mid expiratory flow, *FU* Follow-up, *SD* Standard Deviation, *BMI* Body mass index, *hs-CRP* high sensitive C-reactive protein

*Included 1747 subjects (87.8%) who participated in PneumoLaus baseline

### Association between ferritin and spirometric values

**Fig. 2:**
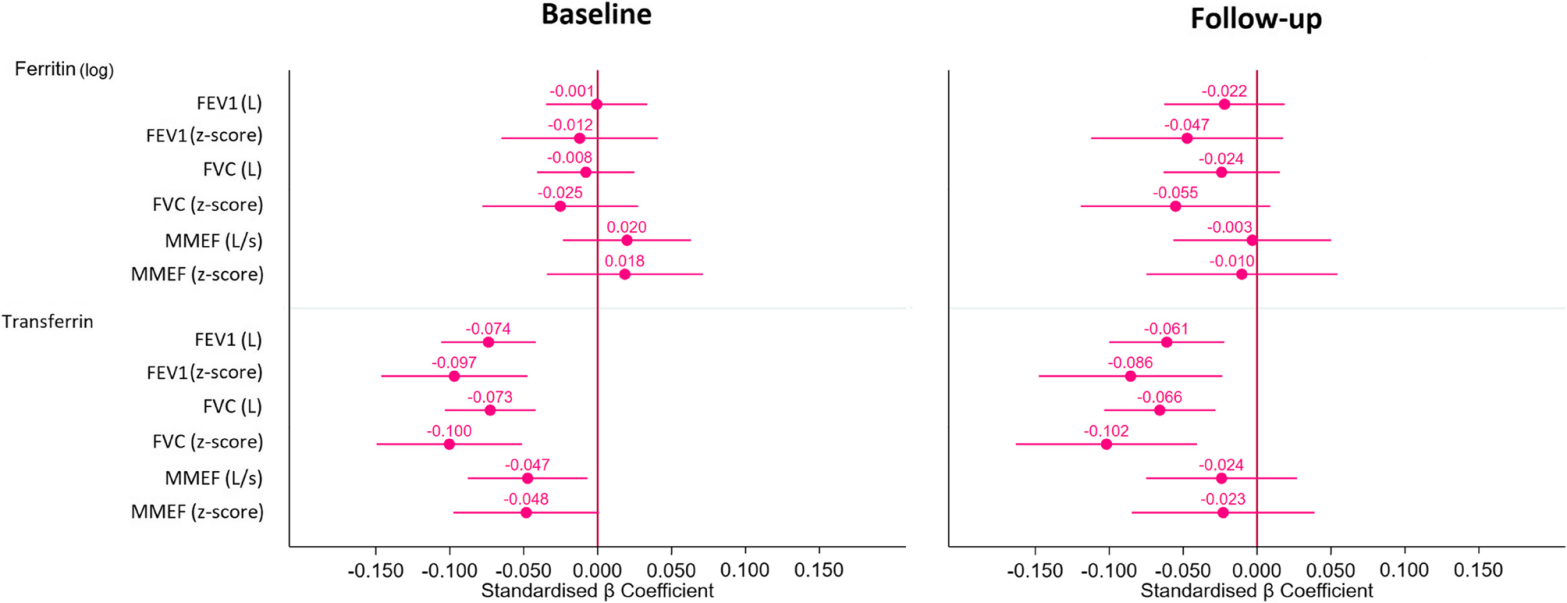
Adjusted associations between iron metabolism markers and spirometric values. FEV1 = Forced expiratory volume in 1 s; FVC = Forced vital capacity; MMEF = maximal mid expiratory flow; L = Litre; L/s = Litre/second. Statistical adjusted analysis conducted using multivariable linear regression adjusting for age, sex, body mass index (continuous), educational level and smoking status, ferritin if transferrin was analysed and transferrin if ferritin was analysed. Ferritin and transferrin were adjusted according to the BRINDA correction. Ferritin was log corrected. Bars represent 99% confidence intervals

**Fig. 3:**
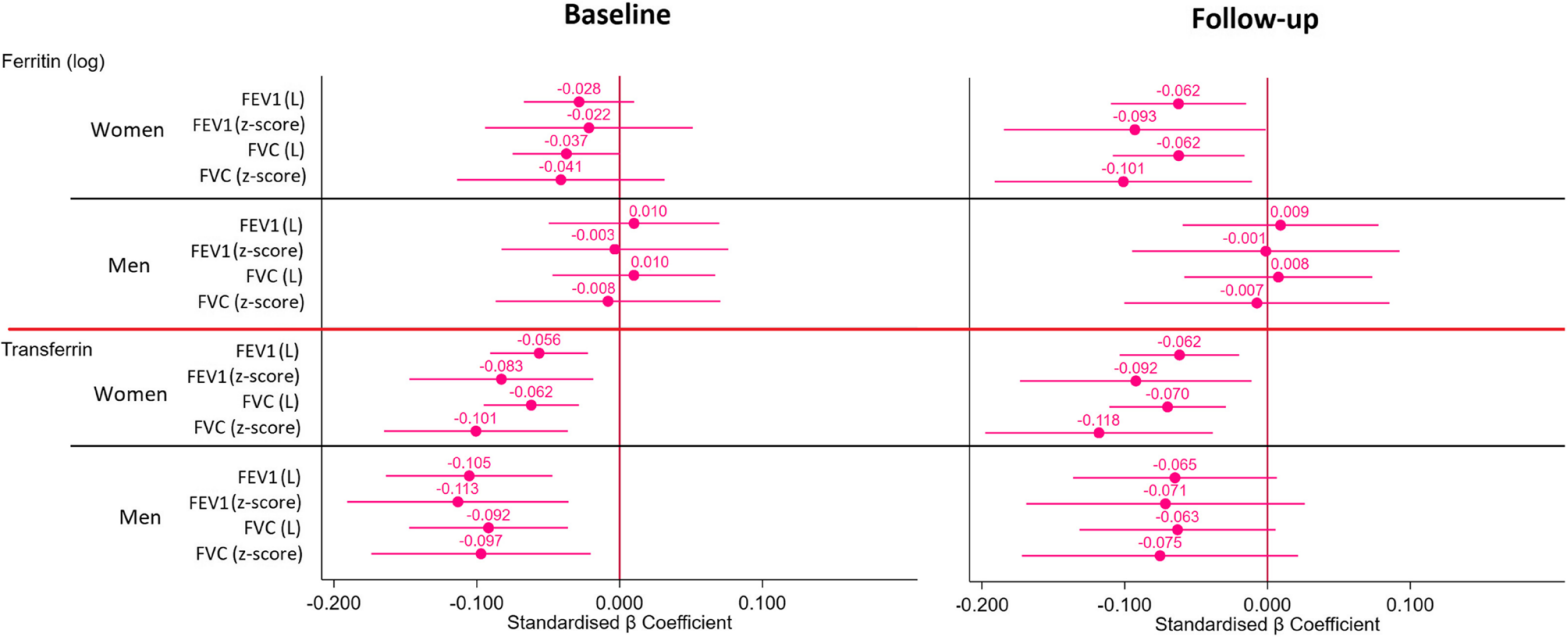
Adjusted associations between iron metabolism markers and spirometric values by sex. FEV1 = Forced expiratory volume in 1 s; FVC = Forced vital capacity; MMEF = maximal mid expiratory flow; L = Litre; L/s = Litre/second. Statistical adjusted analysis conducted using multivariable linear regression adjusting for age, body mass index (continuous), educational level and smoking status, ferritin if transferrin was analysed and transferrin if ferritin was analysed. Ferritin and transferrin were adjusted according to the BRINDA correction. Ferritin was log corrected. Bars represent 99% confidence intervals

Associations of ferritin levels with spirometric values in bivariate and multivariable analyses are summarised in Tables 2 and 3; Figs. 2 and 3 and Table S3, S4 in the supporting information.

**Table 2:** Unadjusted association between ferritin or transferrin and spirometric values using pearson correlation, overall and by sex

	Ferritin (log)			Transferrin		
Baseline	Overall (*n* = 3102)	Women (*n* = 1736)	Men (*n* = 1366)	Overall (*n* = 3102)	Women (*n* = 1736)	Men (*n* = 1366)
FEV1 (Litre), [CI99]	+ 0.199** [+ 0.155;+0.244]	−0.129** [−0.195;−0.064]	+ 0.063 [−0.008;+0.135]	−0.088 [−0.133;−0.044]	−0.082* [−0.147;−0.017]	−0.111** [−0.179;−0.043]
FEV1 (z-score), [CI99]	−0.004 [−0.050;+0.043]	+ 0.002 [−0.064;+0.067]	+ 0.016 [−0.062;+0.094]	−0.098** [−0.144;−0.052]	−0.082** [−0.145;−0.019]	−0.119** [−0.187;−0.052]
FVC (Litre), [CI99]	+ 0.216** [+ 0.171;+0.260]	−0.132** [−0.201;−0.064]	+ 0.056 [−0.014;+0.126]	−0.093** [−0.137;−0.049]	−0.096** [−0.164;−0.029]	−0.118** [−0.185;−0.050]
FVC (z-score), [CI99]	−0.019 [−0.70;+0.31]	−0.007 [−0.073;+0.059]	−0.000 [−0.077;+0.077]	−0.108** [−0.156;−0.059]	−0.102** [−0.165;−0.39]	−0.118** [−0.190;−0.046]
MMEF (Litre/s), [CI99]	+ 0.126** [+ 0.081;+0.171]	−0.081** [−0.143;−0.019]	+ 0.060 [−0.009;+0.128]	−0.050* [−0.094;−0.006]	−0.032 [−0.095;+0.031]	−0.060 [−0.129;+0.010]
MMEF (z-score), [CI99]	+ 0.043 [−0.004;+0.090]	+ 0.027 [−0.038;+0.093]	+ 0.029 [−0.041;+0.100]	−0.047 [−0.095;+0.001]	−0.027 [−0.089;+0.035]	−0.069* [−0.137;−0.001]
Follow-up	Overall (*n* = 1989)	Women (*n* = 1101)	Men (*n* = 888)	Overall (*n* = 1989)	Women (*n* = 1101)	Men (*n* = 888)
FEV1 (Litre), [CI99]	+ 0.194** [+ 0.142;+0.248]	−0.096* [−0.173;−0.020]	+ 0.071 [−0.009;+0.153]	−0.087** [−0.145:−0.029]	−0.089* [−0.165;−0.012]	−0.072 [−0.155;+0.012]
FEV1 (z-score), [CI99]	−0.029 [−0.086;+0.029]	−0.055 [−0.131;+0.021]	+ 0.015 [−0.069;+0.098]	−0.084** [−0.143;−0.025]	−0.074 [−0.149;+0.001]	−0.099* [−0.184;−0.013]
FVC (Litre), [CI99]	+ 0.203** [+ 0.151;+0.255]	−0.097* [−0.179;−0.015]	+ 0.063 [−0.018;+0.145]	−0.099** [−0.156;−0.042]	−0.111** [−0.189;−0.034]	+ 0.083* [−0.166;−0.002]
FVC (z-score), [CI99]	−0.045 [−0.107;+0.165]	−0.061 [−0.140;+0.018]	−0.003 [−0.090;+0.095]	−0.107** [−0.166;−0.047]	−0.108** [−0.190;−0.027]	−0.111** [−0.195;−0.027]
MMEF (Litre/s), [CI99]	+ 0.132** [+ 0.072;+0.192]	−0.055 [−0.131;+0.020]	+ 0.065 [−0.028;+0.157]	−0.031 [−0.088;+0.026]	−0.015 [−0.090;+0.061]	−0.017 [−0.102;+0.067]
MMEF (z-score), [CI99]	+ 0.021 [−0.037;+0.078]	−0.015 [−0.091;+0.062]	+ 0.019 [−0.067;+0.106]	−0.019 [−0.076;+0.038]	−0.000 [−0.071;+0.071]	−0.037 [−0.120;+0.047]

*FEV1* Forced expiratory volume in 1 s, *FVC* Forced vital capacity, *MMEF* maximal mid expiratory flow

Ferritin and transferrin were adjusted according to the BRINDA correction. Ferritin was log corrected. * = *p* < 0.01; ** = *p* < 0.001

**Table 3:** Association between spirometric values and blood test metabolism in follow-up (2018-2021) using transferrin saturation

	Ferritin (log)	Transferrin	TSAT (%)	
Follow-up	Standardised β Coefficient adjusted	Standardised β Coefficient adjusted	Pearson correlation	Standardised β Coefficient adjusted
FEV1 (Litre), [CI99]	−0.025 [−0.067;+0.017]	−0.058** [−0.099;−0.018]	+0.074** [+0.019;+0.130]	+0.011 [−0.028;+0.051]
FEV1 (z-score), [CI99]	−0.051 [−0.118;+0.016]	−0.082** [−0.146;−0.079]	+0.022 [−0.035;+0.080]	+0.015 [−0.048;+0.079]
FVC (Litre), [CI99]	−0.029 [−0.070;+0.011]	−0.061** [−0.099;−0.022]	+0.093** [+0.037;+0.149]	+0.021 [−0.018;+0.059]
FVC (z-score), [CI99]	−0.063 [−0.129;+0.003]	−0.094** [−0.157;−0.030]	+0.043 [−0.012;+0.098]	+0.032 [−0.031;+0.094]
MMEF (Litre/s), [CI99]	−0.001 [−0.056;+0.054]	−0.026 [−0.079;+0.027]	+0.019 [−0.039;+0.077]	−0.009 [−0.061;+0.043]
MMEF (z-score), [CI99]	−0.008 [−0.075;+0.058]	−0.025 [−0.089;+0.039]	−0.004 [−0.060;+0.052]	−0.008 [−0.072;+0.055]

Unadjusted analysis is performed using a Pearson correlation. Statistical adjusted analysis conducted using multivariable linear regression adjusting for age, sex, body mass index (continuous), educational level, smoking status and TSAT and ferritin if transferrin was analysed, transferrin and ferritin if TSAT was analysed, TSAT and transferrin if ferritin was analysed. Ferritin, transferrin and TSAT were adjusted according to the BRINDA correction. Ferritin was log corrected

*FEV1* Forced expiratory volume in 1 s, *FVC* Forced vital capacity, *MMEF* maximal mid expiratory flow, *TSAT* Transferrin saturation

* = *p* < 0.01; ** = *p* < 0.001

In both surveys, ferritin was positively associated with FEV1, FVC and MMEF for absolute values in the bivariate model. When stratified by sex, we found a negative association between ferritin and absolute FEV1, FVC and MMEF in women (baseline analysis only for MMEF), but no association in men. The negative association between ferritin and FEV1 and FVC was only present in the FU analysis. We found no associations between ferritin and spirometric values in z-score analyses even after adjustment in all subjects. When stratified by sex in the adjusting model, a negative association with FEV1 and FVC (in absolute volume and in z-score) was found only in women at FU (Fig. 3 and Table S3). This negative association remained by adding TSAT to the adjusted model (Table S4).

### Association between transferrin and spirometric values

**Fig. 4:**
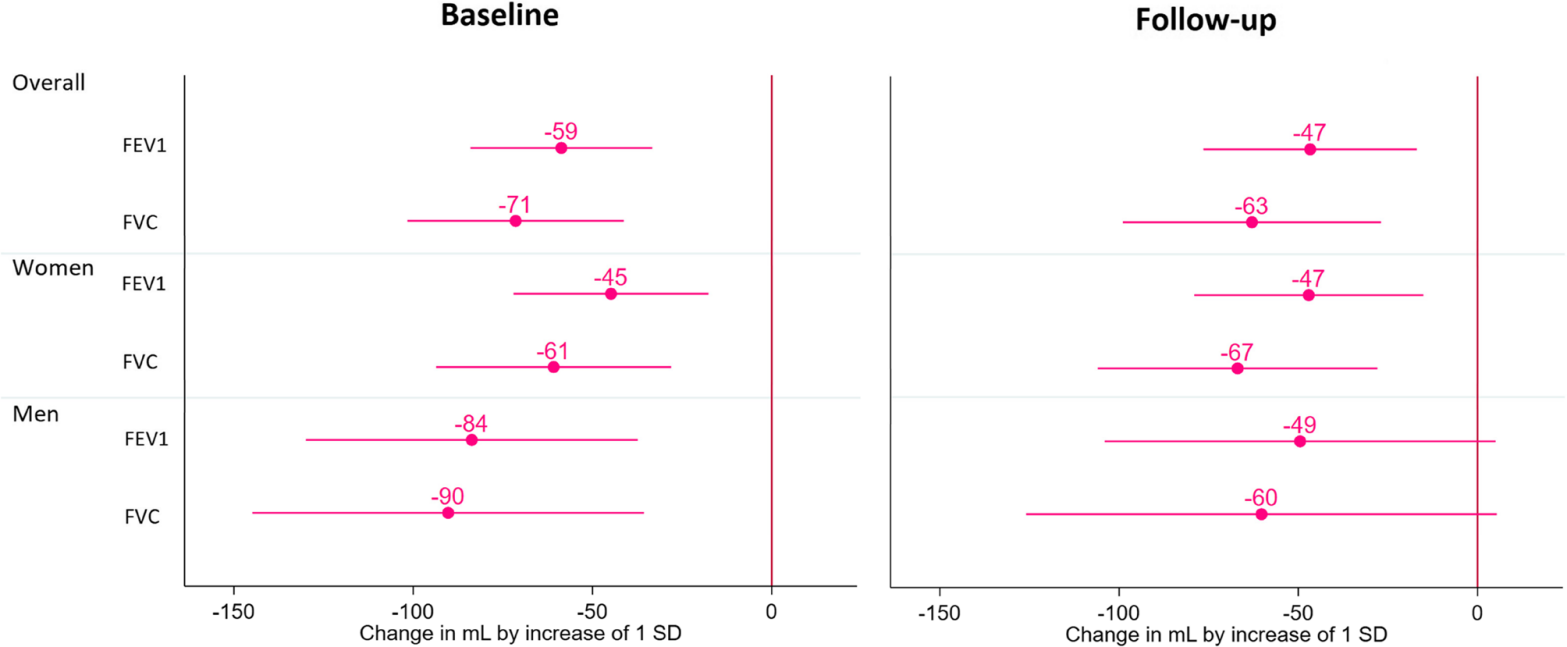
Adjusted spirometric absolute volume change per standard deviation of transferrin, overall and by sex. FEV1 = Forced expiratory volume in 1 s; FVC = Forced vital capacity. Statistical adjusted analysis conducted using multivariable linear regression adjusting for age, sex, body mass index (continuous), educational level and smoking status, ferritin if transferrin was analysed and transferrin if ferritin was analysed. Ferritin and transferrin were adjusted according to the BRINDA correction. Ferritin was log corrected. Bars represent 99% confidence intervals

The associations of transferrin with spirometric values in bivariate and multivariable analyses are summarised in Tables 2 and 3; Figs. 2, 3 and 4, Table S3, S4 and Figure S3 in the supporting information.

The FEV1 and FVC in absolute values and in z-score were negatively associated with transferrin level in the baseline bivariate analysis. These negative associations were also present with FEV1 and FVC in the FU bivariate analysis. In our adjusted model, we found negative associations between transferrin level and FEV1 and FVC in absolute and z-score values at baseline. These negative associations were also present between FEV1 and FVC (absolute values and z-scores) at FU analysis (Fig. 2). In women, we found a negative association between FEV1 and FVC with transferrin in both surveys in the multivariable-adjusted model. In men, these negative associations were present only in the baseline survey (Fig. 3). We found a negative association between MMEF and transferrin on bivariate and multivariable analysis, but only for the absolute value at baseline. We found that increasing transferrin by one SD reduced FEV1 by 47 to 59 mL in our cohort, 45 to 47 mL in women and 49 to 84 mL in men. For FVC, increasing transferrin by one SD reduced volume by 63 to 71 mL in our cohort, 61 to 67 mL in women and 60 to 90 mL in men. However, reduced FEV1 and FVC were not significant in men at FU (Fig. 4).

### Association between TSAT and spirometric values

The associations of TSAT with spirometric values in bivariate and multivariable analysis are summarised in Table 3, S4 and Figure S1 in the supporting information. The TSAT was positively associated with FEV1 and FVC only for absolute values in bivariate analysis. We found no association between TSAT and spirometric values in our adjusted model, including when we stratified by sex. The negative association between transferrin levels described above and FEV1 or FVC (in absolute value and z-score) persisted in adjusted model, including TSAT.

## Discussion

Our study suggests a negative association between transferrin levels and spirometric values. The associations between ferritin and spirometric values appeared to be less important, more complex and influenced by sex: a positive association between ferritin and lung volume only in absolute values and in the unadjusted model, with a negative association between ferritin and lung volume in women only, and no reproducible association in our adjusted model.

We acknowledge that the associations observed were generally weak, with all coefficients of correlation *r* < 0.250. Similarly, with standardised β coefficients ranging from − 0.050 to −0.150 (Table S3 in the supporting information), a change of one standard deviation in transferrin therefore modifies less than 0.150 standard deviations in FEV1 or FVC. This suggests that the contributions of iron metabolism to lung function values in a general urban population are modest, especially after adjustment for other confounders, despite the statistically significant associations. Nevertheless, the repeated significant associations between spirometric values and blood values across two surveys and other studies support the plausibility to the underlying pathophysiological mechanisms [1, 4, 6]. In our opinion, clinical interpretation is limited, and such associations can only be used for population-level analysis.

We did not identify a systematic association between ferritin and spirometric values. We found a non-significant negative standardised β coefficient at −0.001 at baseline and − 0.022 at FU for the association between ferritin and FEV1, contrasting with a positive β coefficient of + 0.023 found by Lee et al. on FEV1 and a positive β coefficient of + 0.081 described by Yu et al. [4, 6]. We found no association between ferritin and age-adjusted FEV1 or FVC decrease in contrast to Lee et al. [1]. This may be due to the BRINDA correction, which includes an adjustment for hs-CRP (this corrects for the hs-CRP-associated increase in ferritin). Positive associations between spirometry values and ferritin levels were only observed in our cohort for absolute values and in the unadjusted model, with r levels similar to those reported by Ghio et al. (r from 0.101 to 0.152, r from 0.194 to 0.216 in our study) [7]. These positive associations likely reflect the known association between male sex and higher absolute lung volumes, as described in Table S1 in the supporting information. For absolute lung volumes, this association became negative in the women’s group, which may reflect an age-related confounding factor (younger women have lower ferritin). These factors could decrease the lung volume. Using z-score spirometry values, which take sex differences into account, we observed only few very weak and non-systematic associations. Ferritin levels increase during the perimenopausal period and after menopause in women [22]. This change in iron status after menopause could explain the negative association observed only in women at follow-up. Newly elevated ferritin levels in menopausal women with low oestrogen and progesterone levels could suggest an interaction between oestrogen and/or progesterone with possible cellular iron overload resulting in pulmonary injury. A possible explanation is that cellular iron overload would not occur in the presence of sex hormones. Consequently, biological males and menstruating individuals would be minimally affected by iron overload. The possible link between iron status and hormonal status merits further study. The lack of an adjusted association between BRINDA-adjusted ferritinemia and reduced lung function suggests that elevated ferritin levels may indicate underlying inflammation and adversely affect lung function. Previously described associations between ferritin without the BRINDA correction and lung volumes were also possibly influenced by systemic inflammation inducing changes in transferrin, which appears to have a more direct association with lung volumes. However, the weak negative association between ferritin levels and FVC found only at FU could suggest a negative association present only in postmenopausal women (given the higher age at FU), as suggested by Kim et al. [2].

We observed a weak association between possible iron deficiency expressed by elevated transferrin and lower FEV1 and FVC. This negative association was stronger than previously described by Yu et al. (standardised β coefficient between − 0.061 to −0.102 versus − 0.036 and − 0.039 in Yu et al.) [4]. The presence of this association with transferrin, rather than ferritin, is likely due to its increased production in response to iron deficiency and reflects the actual lack of available iron in the body. This lack of available iron may affect iron-dependent enzymes that are essential for mitochondrial activity and cellular production. Low availability of iron may induce a reduction in the calibre of the large airways, resulting in increased airflow resistance and resulting in lower FEV1 and FVC. In addition, a lack of available iron may alter extracellular remodelling, affecting lung compliance. As suggested by Brigham et al., the lung tissue’s need for iron may lead to lower lung volumes [3]. Given the weak standardised β coefficients (−0.050 to −0.150), this effect is however marginal. Furthermore, the lack of biological availability of iron did not appear to affect the small airways, as indicated by the weak association (*r*>−0.060, standardised β coefficient>−0.050) with MMEF, observed only at baseline in absolute value. In the study of Ozdemir A et al. on β-thalassemia major patients, the authors did not observe a significant differences for MMEF in high versus low ferritin groups [5]. The absence of a significant association with FU transferrin in men is probably due to a lack of power (*n* = 888).

The impact in absolute volume was modest, given the decrease in FEV1 of 47 to 59mL and FVC of 63 to 71mL per 1-SD increase of transferrin. The decrease in FEV1 and FVC per 1-SD of transferrin seemed higher in men than in women but was not consistently significant at FU, likely due to a lack of power (*n* = 888). Furthermore, the interpretation of absolute volume changes in FEV1 and FVC by transferrin is limited for several reasons. The mean difference in absolute volume between men and women (0.96 L for FEV1 and 1.31 L for FVC) increases the standard deviation of absolute volumes when these volumes are analysed together. The difference in the decrease in FEV1 and FVC between men and women could represent the difference in standard deviation in absolute volume between men and women, where the standard deviation is lower in women than in men. In addition, the standard deviation of FEV1 and FVC increases with age, which may increase the absolute volume change in an older population. Consequently, the interpretation of absolute volume difference per 1-SD increase of transferrin should be interpreted with caution.

The positive unadjusted association between TSAT and lung volume observed in our study was weaker than previously reported by Ghio et al. (r from 0.222 to 0.245 for Ghio et al., r from 0.074 to 0.093 in our study) [7]. The BRINDA correction utilised in our analysis may explain this difference. After adjusting for other parameters related to iron metabolism and contrasting with findings of Brigham et al. [3], we found that TSAT was not associated with spirometric values, possibly related to the BRINDA correction and adjustment for hs-CRP. Association between TSAT and lung function was not related to biomarkers of inflammation and iron metabolism. Even after adjustment for TSAT, there was still a negative association between transferrin levels and FEV1 and FVC.

### Strengths and limitations

The main strength of our study lies in the comprehensive data collection, with two blood and spirometric measurements performed four years apart in the general population. We analysed ferritin and transferrin using the BRINDA adjustments to correct for the impact of inflammation on the iron metabolism.

A limitation of our study is a cohort with a majority of participants of Caucasian ethnicity (> 97% of participants), which may limit the external validity of our findings to other ethnical populations. Second, our FU occurred during the COVID-19 pandemic, which led to an interruption in data acquisition, particularly spirometry. As a result, we were unable to perform spirometric monitoring on several subjects, leading to reduced number of participants at FU. Third, due to the loss of 1,730 subjects, the follow-up population may be healthier than at baseline. This loss of subjects could reduce the strength of the associations observed during the follow-up period. Because of our cohort analysis, the very sickest individuals may not have been included in the cohort and consequently in our analysis. This might also have reduced the strength of the associations. Fourth, during cohort data collection, subjects were told not to come to the data centre if they had fever. As a result, subjects with more pronounced inflammation possibly did not perform the survey and this could limit our external inferences for people with high levels of chronic inflammation. Fifth, the soluble receptor of transferrin was not measured, and we therefore could not analyse the possible link to this blood value. Sixth, we have not included some cofounding factors such as diet, which is closely associated to iron status, hormone replacement therapy or hormonal status given the suspicion of a hormonal effect, physical activity, anaemia, and iron replacement therapy.

## Conclusion

In this population-based cohort, we observed no reproductible association between ferritin level and spirometric values. A weak negative association remained possible only in slightly older women with FEV1 and FVC. Transferrin level was negatively associated with FEV1 and FVC, and marginally with the MMEF, suggesting that iron metabolism, particularly the requirement for iron in the organism, is linked to reduced lung volumes. The clinical relevance of the negative association with iron metabolism and lung function, in particular iron deficiency, needs to be further investigated in the future.

## Supplementary Information

Supplementary Material 1.

## Data Availability

All data relevant to this work are included in the manuscript and its supplementary information files. However, full access to the CoLaus|PsyCoLaus and PneumoLaus datasets is restricted due to the inclusion of sensitive personal data. In accordance with Swiss privacy laws and the requirements of the Canton of Vaud’s Ethics Committee, sharing of these datasets in their entirety is not permitted and would be a violation of the Swiss legislation with respect to privacy protection. Researchers affiliated to research institution who fulfil the CoLaus|PsyCoLaus data sharing policy can request anonymised, coded datasets that do not allow identification of participants. Such requests can be addressed to the CoLaus|PsyCoLaus Datacenter (CHUV, Lausanne, Switzerland) via email: research.psycolaus@chuv.ch. Requests involving only baseline data are reviewed by the baseline (local) scientific committee, while those involving follow-up data are evaluated by the follow-up (multicentric) scientific committee. Full access guidelines for gaining access to the CoLaus|PsyCoLaus data are available at: www.colaus-psycolaus.ch/professionals/how-to-collaborate.

## Abbreviations

BMI: Body mass index
BRINDA: Biomarkers Reflecting Inflammation and Nutrition Determinants of Anemia
FEV1: Forced Expiratory Volume in 1 s
FU: Follow-up
FVC: Forced Vital Capacity
hs-CRP: high sensitive C-reactive protein
MMEF: Maximal mid expiratory flow
TSAT: Transferrin saturation
