# Supplementary material for: Association between BRINDA-corrected iron metabolism and lung function in the community: the CoLaus|PneumoLaus study
Source: BMC Pulm Med. 2025 Sep 30;25:438. doi: 10.1186/s12890-025-03810-x (PMC12486500; doi:10.1186/s12890-025-03810-x)
Supplement: Supplementary file 1 — Supplementary Material 1. [file 12890_2025_3810_MOESM1_ESM.docx]

**SUPPLEMENTARIES**

**Table S1:** Spirometric and iron metabolism characteristics at baseline and follow-up, by sex

|  | Baseline | | | Follow-up | | |
| --- | --- | --- | --- | --- | --- | --- |
|  | Female (N=1736) | Male (N=1366) | p-value | Female (N=1101) | Male (N=888) | p-value |
| FEV1, Litre (SD) | 2.41 (0.53) | 3.37 (0.75) | <0.001 | 2.33 (0.49) | 3.31 (0.69) | <0.001 |
| FVC, Litre (SD) | 3.10 (0.62) | 4.41 (0.87) | <0.001 | 3.01 (0.60) | 4.31 (0.82) | <0.001 |
| MMEF, Litre/s (SD) | 2.21 (0.84) | 3.00 (1.21) | <0.001 | 2.15 (0.79) | 2.97 (1.13) | <0.001 |
| Ferritin in µg/L, mean (SD) | 135.6 (95.7) | 244.9 (194.7) | <0.001 | 136.8 (84.7) | 225.6 (167.2) | <0.001 |
| Ferritin in µg/L, median (Q1-Q3) | 117 (74 – 176.5) | 194 (127 – 304) | <0.001^1^ | 123 (77 – 173) | 185 (119 – 280) | <0.001^1^ |
| TSAT, mean (SD) | - | - | - | 28.7 (8.8) | 31.1 (10.2) | <0.001 |

FEV1 = Forced expiratory volume in 1 second; FVC = Forced vital capacity; MMEF = mid-maximal expiratory flow; TSAT = Transferrin saturation

1: using Mann-whitney test

**Table S2:** Unadjusted association between ferritin or transferrin and spirometric values using spearman rank correlation, overall and by sex

|  | Ferritin | | | Transferrin | | |
| --- | --- | --- | --- | --- | --- | --- |
|  | Overall (n=3102) | Women (n=1736) | Men (n=1366) | Overall (n=1989) | Women (n=1101) | Men (n=888) |
| Baseline |  |  |  |  |  |  |
| FEV1 (Litre) | +0.213** | -0.120** | +0.058 | -0.098** | -0.104** | -0.125** |
| FEV1 (z-score) | -0.002 | +0.004 | +0.011 | -0.105** | -0.089** | -0.127** |
| FVC (Litre) | +0.233** | -0.117** | +0.047 | -0.099** | -0.117** | -0.132** |
| FVC (z-score) | -0.019 | -0.002 | -0.013 | -0.113** | -0.104** | -0.127** |
| MMEF (Litre/s) | +0.124** | -0.079* | +0.060 | -0.068** | -0.049 | -0.077* |
| MMEF (z-score) | +0.043 | +0.024 | +0.038 | -0.056* | -0.037 | -0.077* |
| Follow-up |  |  |  |  |  |  |
| FEV1 (Litre) | +0.198** | -0.101** | +0.064 | -0.091** | -0.086* | -0.074 |
| FEV1 (z-score) | -0.029 | -0.055 | +0.016 | -0.082** | -0.059 | -0.113** |
| FVC (Litre) | +0.213** | -0.093* | +0.049 | -0.102** | -0.116** | -0.086 |
| FVC (z-score) | -0.044 | -0.055 | -0.004 | -0.107** | -0.098* | -0.125** |
| MMEF (Litre/s) | +0.123** | -0.069 | +0.068 | -0.036 | -0.010 | -0.019 |
| MMEF (z-score) | +0.016 | -0.025 | +0.033 | -0.021 | -0.011 | -0.051 |

FEV1 = Forced expiratory volume in 1 second; FVC = Forced vital capacity ; MMEF = mid-maximal expiratory flow; protein

Ferritin and transferrin were adjusted according to the BRINDA correction.

* = p<0.01; ** = p<0.001

**Table S3:** Adjusted association between ferritin or transferrin and spirometric values using standardised β coefficient multivariable linear regression, overall and by sex

|  | Ferritin (log) | | | Transferrin | | |
| --- | --- | --- | --- | --- | --- | --- |
| Baseline | Overall (n=3102) | Women (n=1736) | Men (n=1366) | Overall (n=3102) | Women (n=1736) | Men (n=1366) |
| FEV1 (Litre), [CI99] | -0.001  [-0.035;+0.034] | -0.028  [-0.067;+0.010] | +0.010  [-0.050;+0.070] | -0.074**  [-0.106;-0.042] | -0.056**  [-0.091;-0.022] | -0.105**  [-0.164;-0.047] |
| FEV1 (z-score), [CI99] | -0.012  [-0.065;+0.041] | -0.022  [-0.094;+0.051] | -0.003  [-0.083;+0.076] | -0.097**  [-0.146;-0.048] | -0.083**  [-0.147;-0.018] | -0.113**  [-0.191;-0.036] |
| FVC (Litre), [CI99] | -0.008  [-0.041;+0.025] | -0.037  [-0.075;+0.001] | +0.010  [-.0.47;+0.067] | -0.073**  [-0.103;-0.042] | -0.062**  [-0.095;-0.029] | -0.092**  [-0.147;-0.036] |
| FVC (z-score), [CI99] | -0.025  [-0.078;+0.027] | -0.041  [-0.114;+0.032] | -0.008  [-0.87;+0.71] | -0.100**  [-0.150;-0.051] | -0.101**  [-0.165;-0.036] | -0.097*  [-0.174;-0.020] |
| MMEF (Litre/s), [CI99] | +0.020  [-0.024;+0.063] | +0.004  [-0.045;+0.054] | +0.014  [-0.062;+0.090] | -0.047*  [-0.088;-0.007] | -0.026  [-0.070;+0.017] | -0.086*  [-0.160;-0.012] |
| MMEF (z-score), [CI99] | +0.019  [-0.034;+0.071] | +0.025  [-0.046;+0.097] | +0.011  [-0.069;+0.093] | -0.048  [-0.098;+0.001] | -0.025  [-0.088;+0.038] | -0.076  [-0.155;+0.003] |
| Follow-up | Overall (n=1989) | Women (n=1101) | Men (n=888) | Overall (n=1989) | Women (n=1101) | Men (n=888) |
| FEV1 (Litre), [CI99] | -0.022  [-0.063;+0.019] | -0.062*  [-0.110;-0.015] | +0.009  [-0.059;+0.078] | -0.061**  [-0.100:-0.022] | -0.062*  [-0.103;-0.020] | -0.065  [-0.136;+0.007] |
| FEV1 (z-score), [CI99] | -0.047  [-0.112;+0.018] | -0.093*  [-0.185;-0.001] | +0.001  [-0.095;+0.092] | -0.086**  [-0.148;-0.024] | -0.092*  [-0.179;-0.011] | -0.071  [-0.169;+0.026] |
| FVC (Litre), [CI99] | -0.024  [-0.063;+0.015] | -0.062**  [-0.108;-0.016] | +0.008  [-0.058;+0.073] | -0.066**  [-0.104;-0.028] | -0.070**  [-0.111;-0.029] | +0.063  [-0.132;+0.006] |
| FVC (z-score), [CI99] | -0.055  [-0.119;+0.009] | -0.101*  [-0.191;-0.011] | -0.007  [-0.100;+0.085] | -0.102**  [-0.163;-0.041] | -0.118**  [-0.198;-0.038] | -0.075  [-0.172;-0.021] |
| MMEF (Litre/s), [CI99] | -0.003  [-0.057;+0.050] | -0.035  [-0.097;+0.027] | +0.017  [-0.074;+0.108] | -0.024  [-0.075;+0.027] | -0.021  [-0.076;+0.033] | -0.034  [-0.129;+0.061] |
| MMEF (z-score), [CI99] | +0.010  [-0.075;+0.054] | -0.030  [-0.119;+0.059] | +0.012  [-0.085;+0.108] | -0.023  [-0.085;+0.039] | -0.017  [-0.095;+0.062] | -0.026  [-0.126;+0.075] |

FEV1 = Forced expiratory volume in 1 second; FVC = Forced vital capacity; MMEF = mid-maximal expiratory flow.

Statistical adjusted analysis conducted using multivariable linear regression adjusting for age, sex, body mass index (continuous), educational level and smoking status, ferritin if transferrin was analysed and transferrin if ferritin was analysed. Ferritin and transferrin were adjusted according to the BRINDA correction. Ferritin was log corrected. * = p<0.01; ** = p<0.001

**Table S4.** Association between spirometric values and blood test metabolism in Follow-up (2018-2021) using transferrin saturation by sex

|  | Ferritin (log) | | Transferrin | | TSAT (%) | | | |
| --- | --- | --- | --- | --- | --- | --- | --- | --- |
| Follow-up | **Standardised β Coefficient adjusted** | **Standardised β Coefficient adjusted** | **Standardised β Coefficient adjusted** | **Standardised β Coefficient adjusted** | **Pearson correlation** | **Pearson correlation** | **Standardised β Coefficient adjusted** | **Standardised β Coefficient adjusted** |
|  | **Women (n=1101)** | **Men (n=888)** | **Women (n=1101)** | **Men (n=888)** | **Women (n=1101)** | **Men (n=888)** | **Women (n=1101)** | **Men (n=888)** |
| FEV1 (Litre), [CI99] | -0.069**  [-0.118;-0.020] | +0.007  [-0.064;-0.077] | -0.055*  [-0.099;+0.012] | -0.062  [-0.136;+0.011] | +0.041  [-0.038;+0.118] | -0.018  [-0.103;+0.068] | +0.025  [-0.022;+0.072] | +0.009  [-0.055;+0.074] |
| FEV1 (z-score), [CI99] | -0.101*  [-0.196;-0.006] | -0.003  [-0.099;+0.093] | -0.084  [-0.169;+0.000] | -0.070  [-0.170;+0.030] | +0.026  [-0.049;+0.100] | +0.025  [-0.055;+0.107] | +0.030  [-0.062;+0.121] | +0.005  [-0.083;+0.094] |
| FVC (Litre), [CI99] | -0.070**  [-0.118;-0.022] | +0.002  [-0.066;+0.070] | -0.063**  [-0.105;-0.020] | -0.057  [-0.128;-0.013] | +0.055  [-0.022;+0.133] | +0.005  [-0.077;+0.089] | +0.028  [-0.018;+0.075] | +0.023  [-0.039;+0.085] |
| FVC (z-score), [CI99] | -0.111*  [-0.204;-0.018] | -0.015  [-0.110;+0.080] | -0.108**  [-0.191;-0.025] | -0.067  [-0.166;-0.032] | +0.046  [-0.028;+0.118] | +0.054  [-0.029;+0.137] | +0.037  [-0.053 :+0.127] | +0.031  [-0.057;+0.119] |
| MMEF (Litre/s), [CI99] | -0.037  [-0.101;+0.027] | +0.021  [-0.073;+0.114] | -0.019  [-0.076;+0.027] | -0.038  [-0.135;+0.060] | +0.014  [-0.082;+0.079] | -0.044  [-0.131;+0.042] | +0.007  [-0.054 :+0.068] | -0.015  [-0.101 :+0.071] |
| MMEF (z-score), [CI99] | -0.031  [-0.123;+0.061] | +0.017  [-0.082;+0.116] | -0.016  [-0.098;+0.066] | -0.031  [-0.134;+0.072] | -0.011  [-0.089;+0.068] | -0.011  [-0.092;+0.071] | +0.004  [-0.085 :+0.092] | -0.021  [-0.112 :+0.070] |

FEV1 = Forced expiratory volume in 1 second; FVC = Forced vital capacity ; MMEF = mid-maximal expiratory flow; TSAT = Transferrin saturation

Unadjusted analysis is performed using a pearson correlation. Statistical adjusted analysis conducted using multivariable linear regression adjusting for age, body mass index (continuous) , educational level, smoking status and TSAT and ferritin if transferrin was analysed, transferrin and ferritin if TSAT was analysed, TSAT and transferrin if ferritin was analysed. Ferritin, transferrin and TSAT were adjusted according to the BRINDA correction. Ferritin was log corrected.

* = p<0.01; ** = p<0.001

**Figure S1** Association between spirometric values and blood test metabolism in follow-up (2018-2021) using transferrin saturation


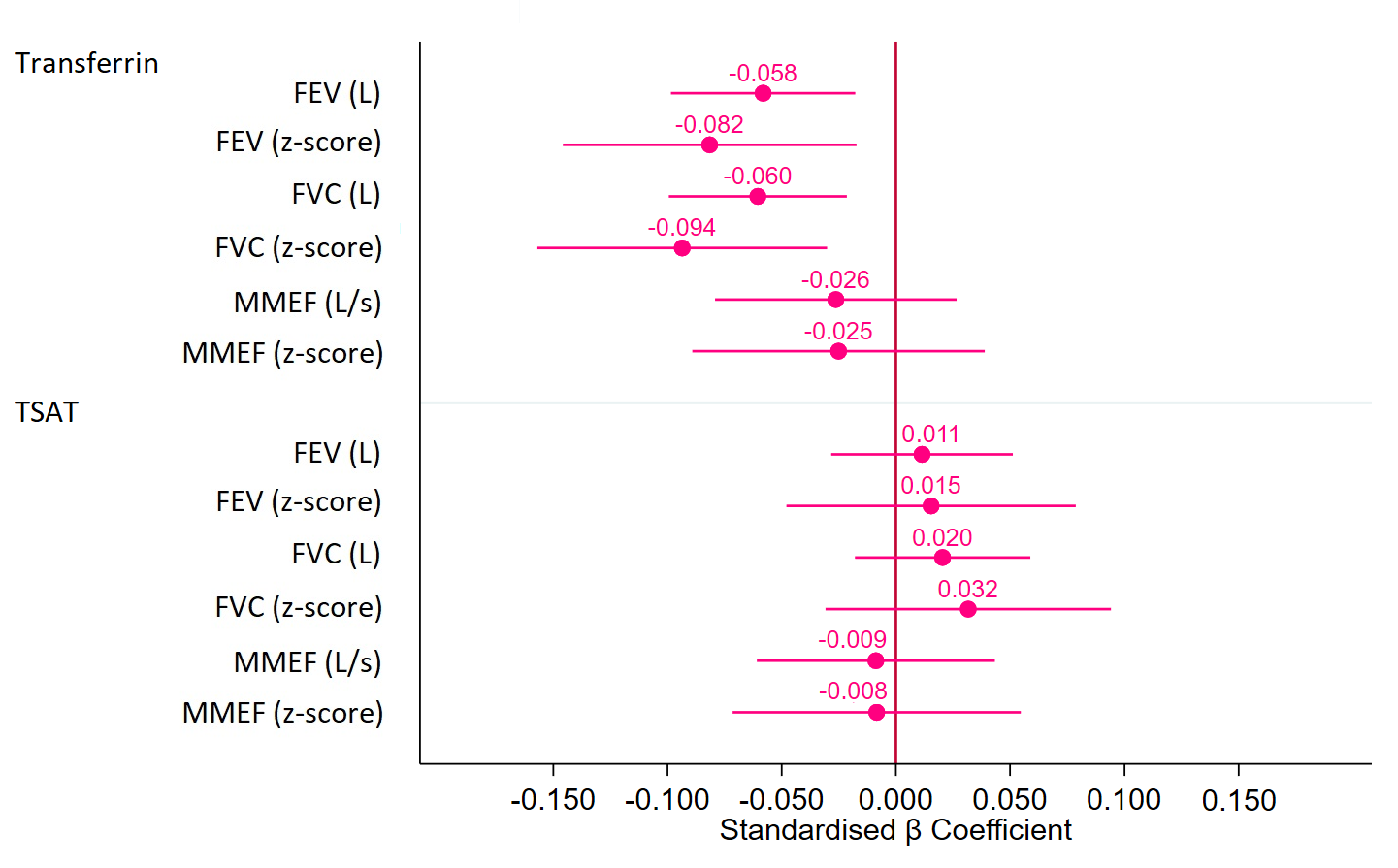


FEV = Forced expiratory volume in 1 second; FVC = Forced vital capacity; MMEF = mid-maximal expiratory flow; L = Litre; L/S = Litre/second

Statistical adjusted analysis conducted using multivariable linear regression adjusting for age, sex, body mass index (continuous) , ferritin, educational level and smoking status, TSAT if transferrin was analysed and transferrin if TSAT was analysed . Ferritin, transferrin and transferrin saturation were adjusted according to the BRINDA correction.
